# Pneumococcal Aetiology and Serotype Distribution in Paediatric Community-Acquired Pneumonia

**DOI:** 10.1371/journal.pone.0089013

**Published:** 2014-02-18

**Authors:** Iris De Schutter, Anne Vergison, David Tuerlinckx, Marc Raes, Julie Smet, Pierre R. Smeesters, Jan Verhaegen, Françoise Mascart, Filip Surmont, Anne Malfroot

**Affiliations:** 1 Department of Pediatric Pulmonology, Cystic Fibrosis Clinic and Pediatric Infectious Diseases, Universitair Ziekenhuis Brussel (UZ Brussel), Brussels, Belgium; 2 Pediatric Infectious Disease Department, Infection Control and Hospital Epidemiology Unit, Université Libre de Bruxelles-Hôpital Universitaire des Enfants Reine Fabiola, Brussels, Belgium; 3 Department of Pediatrics, Université Catholique Louvain, University Hospital Mont-Godinne, Yvoir, Belgium; 4 Department of Pediatrics, Virga Jesse Hospital, Hasselt, Belgium; 5 Immunobiology Clinic, Hôpital Erasme, Brussels, Belgium; 6 Laboratory of Vaccinology and Mucosal Immunity, Université Libre de Bruxelles, Brussels, Belgium; 7 Bacterial Genetics and Physiology Laboratory, Université Libre de Bruxelles, Gosselies, Belgium; 8 Murdoch Childrens Research Institute, Melbourne, Australia; 9 Department of Laboratory Medicine-Microbiology, Pneumococcal Reference Laboratory, Universitair Ziekenhuis Katholieke Universiteit Leuven, Leuven, Belgium; 10 Pfizer Inc., Brussels, Belgium; Centers for Disease Control & Prevention, United States of America

## Abstract

Community-acquired pneumonia (CAP) is a major cause of morbidity in children. This study estimated the proportion of children with pneumococcal CAP among children hospitalised with CAP in Belgium and describes the causative serotype distribution after implementation of the 7-valent pneumococcal conjugate vaccine. Children 0–14 years hospitalised with X-ray-confirmed CAP were prospectively enrolled in a multicentre observational study. Acute and convalescent blood samples were collected. Pneumococcal aetiology was assessed by conventional methods (blood or pleural fluid cultures with Quellung reaction capsular typing or polymerase chain reaction [PCR] in pleural fluid), and recently developed methods (real-time PCR in blood and World Health Organization-validated serotype-specific serology). A total of 561 children were enrolled. Pneumococcal aetiology was assessed by conventional methods in 539, serology in 171, and real-time PCR in blood in 154. Pneumococcal aetiology was identified in 12.2% (66/539) of the children by conventional methods alone but in 73.9% by the combination of conventional and recently developed methods. The pneumococcal detection rate adjusted for the whole study population was 61.7%. Serotypes 1 (42.3%), 5 (16.0%), and 7F(7A) (12.8%) were predominant. In conclusion, *Streptococcus pneumoniae* remains the predominant bacteria in children hospitalised for CAP in Belgium after implementation of 7-valent pneumococcal conjugate vaccine, with non-vaccine-serotypes accounting for the majority of cases. The use of recently developed methods improves diagnosis of pneumococcal aetiology.

## Introduction

Community-acquired pneumonia (CAP) is a major cause of morbidity and mortality in young children, even in industrialised countries [1]. *Streptococcus pneumoniae* is considered the predominant bacterial pathogen of CAP but aetiological diagnosis in childhood CAP is particularly challenging because lungs are difficult to sample, blood cultures lack sensitivity, and upper respiratory tracts are frequently colonised [1], [2]. Molecular detection techniques and serological assays with higher sensitivity have been developed to improve evaluation of pneumococcal aetiology [3]–[5].

Following the introduction of the 7-valent pneumococcal conjugate vaccine (PCV7; Prevenar, Pfizer, Inc.), hospitalisation rates for childhood CAP decreased in many countries [6], [7]. In Belgium, PCV7 became available in 2004 and, in 2007, universal infant vaccination was implemented with catch-up for children <2 years of age. PCV7 uptake reached 81% in Wallonia and 89% in Flanders in 2008–2009 [8]. The burden of pneumococcal CAP (P-CAP) and causative serotype distribution in Belgian children following the implementation of PCV7 is unknown.

In this study, we evaluated pneumococcal aetiology and serotype distribution in children hospitalised for CAP in Belgium. We used a combination of conventional and additional detection methods (serology and real-time PCR [rtPCR] in blood) to maximise the diagnosis of pneumococcal aetiology.

## Methods

### Ethics statement

The study was conducted in accordance with the Declaration of Helsinki and the study protocol was approved by the ethics committees of all the participating institutions (Text S1). Written informed consent was obtained from the parents before inclusion.

### Study design and patients

This was a prospective observational study conducted in 19 Belgian hospitals between September 1, 2008 and December 31, 2009. Children aged <15 years with radiologically confirmed CAP (i.e., presence of clinical symptoms and signs and chest X-ray findings compatible with CAP [9]) were enrolled. Children with chronic pulmonary diseases, intravenous immunoglobulin treatment, pneumonia diagnosed ≥72 h after hospital admission, or with a relapsing or recurrent CAP episode within 21 days were excluded. Children were managed according to the local hospital’s standard protocol.

The main features of disease, previous antibiotic treatment, and history of pneumococcal vaccination were recorded at inclusion. Patients were considered to have proven P-CAP, suspected P-CAP, or possible P-CAP according to defined criteria (Table 1).

**Table 1:** Criteria used to define proven, suspected, and possible P-CAP.

Disease group	Defined criteria
Proven P-CAP	*S. pneumoniae* isolated from blood or pleural fluid OR PCR in pleural fluid positive for *S. pneumoniae* DNA
Suspected P-CAP	Lobar pneumonia and pleural effusion on chest X-ray AND CRP concentration >40 mg/L at admission AND No bacterial growth on blood or pleural fluid cultures OR PCR in pleural fluid negative for *S. pneumoniae* DNA
Possible P-CAP	All patients not meeting the criteria for proven or suspected P-CAP

CRP, C-reactive protein; P-CAP, pneumococcal community-acquired pneumonia; PCR, polymerase chain reaction.

### Samples and detection methods

At admission, a routine blood sample was collected for blood culture, peripheral blood cell count, and CRP concentration. An additional blood sample was collected for serology and rtPCR. A “convalescent” blood sample was collected 3–4 weeks after admission. When available, pleural fluid was analysed by culture or PCR to detect pneumococcal DNA [10]. Nasopharyngeal aspirates or swabs were collected for the detection of viruses and atypical pathogens only when they were part of the hospital’s routine analysis for childhood CAP.

All routine samples were assessed by the local microbiology laboratories according to their standard protocols. Pneumococcal isolates were sent to the Belgian Reference Laboratory for *S. pneumoniae* (Leuven, Belgium) for capsular serotyping by Quellung reaction as previously described [11].

*S. pneumoniae* serotype-specific serology (SSS). *S. pneumoniae* serotype-specific serology (SSS) was performed for patients for whom at least one pneumococcal microbiological detection method was performed and for whom paired serum samples were available. Available sera from patients with proven and suspected P-CAP were analysed. In addition, a subset of eligible patients with possible P-CAP was randomly selected. Concentrations of IgG and IgA against serotypes 1, 5, 6B(6D), 7F(7A), 9V(9N), 14, 19A, 19F, and 23F capsular polysaccharides were measured using the World Health Organization (WHO)-validated enzyme-linked immunosorbent assay (ELISA) with 22F pre-adsorption as described previously [12].

Briefly, sera pre-adsorbed to pneumococcal cell wall polysaccharide (CWPS) (50 µg/mL; Statens Serum Institute, Copenhagen) and to serotype 22F polysaccharide (10 µg/mL; LGC Promochem) were incubated for 2 h at 37°C, at two different dilutions (1∶10 and 1∶100), in ELISA plates coated during one night at 4°C with purified pneumococcal polysaccharides (10 µg/mL; Merck & Co., American Type Culture Collection). After washing, goat anti-human IgG (alkaline phosphatase-conjugated, Biosource, Paisley, United Kingdom) or IgA (horseradish peroxidase-conjugated, Dako, Heverlee, Belgium) were added before the final step with the chromogenic substrate. Optical densities were measured at 450 nm. Standard curves for IgG were generated by serial dilutions of the standard reference serum 89-SF (Food and Drug Administration, Bethesda, MD) pre-adsorbed to CWPS but not to the 22F polysaccharide as recommended, whereas standard curves for IgA were generated by serial dilutions of a pool of positive sera.

Patients were considered to have a serotype-specific serological response when they had an increase of ≥3-fold between the admission and convalescent sera and an IgG titre ≥0.6 µg/mL or an IgA titre ≥0.6 AU in the convalescent serum. When a response was observed for several serotypes, the serotype with the highest convalescent-to-admission ratio was considered the infecting serotype. Investigators analysing the serological responses were blinded to the bacteriological results.

**Real-time PCR in blood (rtPCR).** rtPCR targeting the pneumococcal autolysin *lytA* was performed in blood samples as previously described [13]. Each specimen was thawed at room temperature for 30 min, of which 200 µL were used for automated DNA extraction by a BioRobot Universal System (QIAGEN) according to the manufacturer’s instructions. The extracted DNA was eluted in a final volume of 200 µl. Of those, 10 µl was mixed with 10 µl 2X Fast PCR mix (Applied Biosystems) containing primers and probes specific to both lytA and ply (duplex assay). The PCR was performed in a 7900 Fast Real-Time PCR System with Fast 96-well Block Module (Applied Biosystems). The procedure includes a 10-min denaturation at 95°C followed by 45 cycles of amplification at 95°C for 15 s and 60°C for 1 min. The endpoint of the assay is the cycle threshold (Ct) and samples with lytA amplification (Ct <45) were considered positive for *S. pneumoniae*. Positive samples were then re-analysed using primers specific to the 13 vaccine serotypes using the same methodology (monoplex assays). Samples positive in the lytA assay but negative in all 13 serotype/serogroup-specific assays were assigned a non-PCV13 type *S. pneumoniae*.

The frequency of CAP episodes with pneumococcal aetiology was calculated by combining all *S. pneumoniae*-positive results obtained with the different detection methods, assuming no false-positive results. The causative serotype for a patient was determined according to the following hierarchy of assays: Quellung reaction on pneumococcal isolates > rtPCR in blood > SSS-IgG > SSS-IgA.

### Calculations and statistical analysis

rtPCR in blood and SSS were performed for a subset of patients from the three patient groups. To determine their distribution in the whole study population, proportions were adjusted to the total number of patients in each group as follows: percentage of proven P-CAP cases + ([number of positive cases in suspected P-CAP group/number of patients tested in suspected P-CAP group] × percentage of suspected P-CAP patients in the study population) + ([number of positive cases in possible P-CAP group/number of patients tested in possible P-CAP group] × percentage of possible P-CAP patients in the study population). The additional diagnostic value of SSS compared to conventional methods was calculated as follows: (number of positive cases determined by SSS – number of positive cases determined by both SSS and conventional methods)/number of patients tested by both SSS and conventional methods. The additional diagnostic value of rtPCR in blood was calculated similarly.

Results were expressed as medians and interquartile ranges (IQR) for quantitative variables and as numbers and proportions for categorical findings. Medians were compared using Kruskal-Wallis test and proportions were compared using chi-square test or Fisher exact test. Tests were two-tailed and considered significant at p<0.05. Calculations were performed with SAS® version 9.2 (SAS Institute, Cary, NC).

## Results

### Patient and disease characteristics

**Figure 1:**
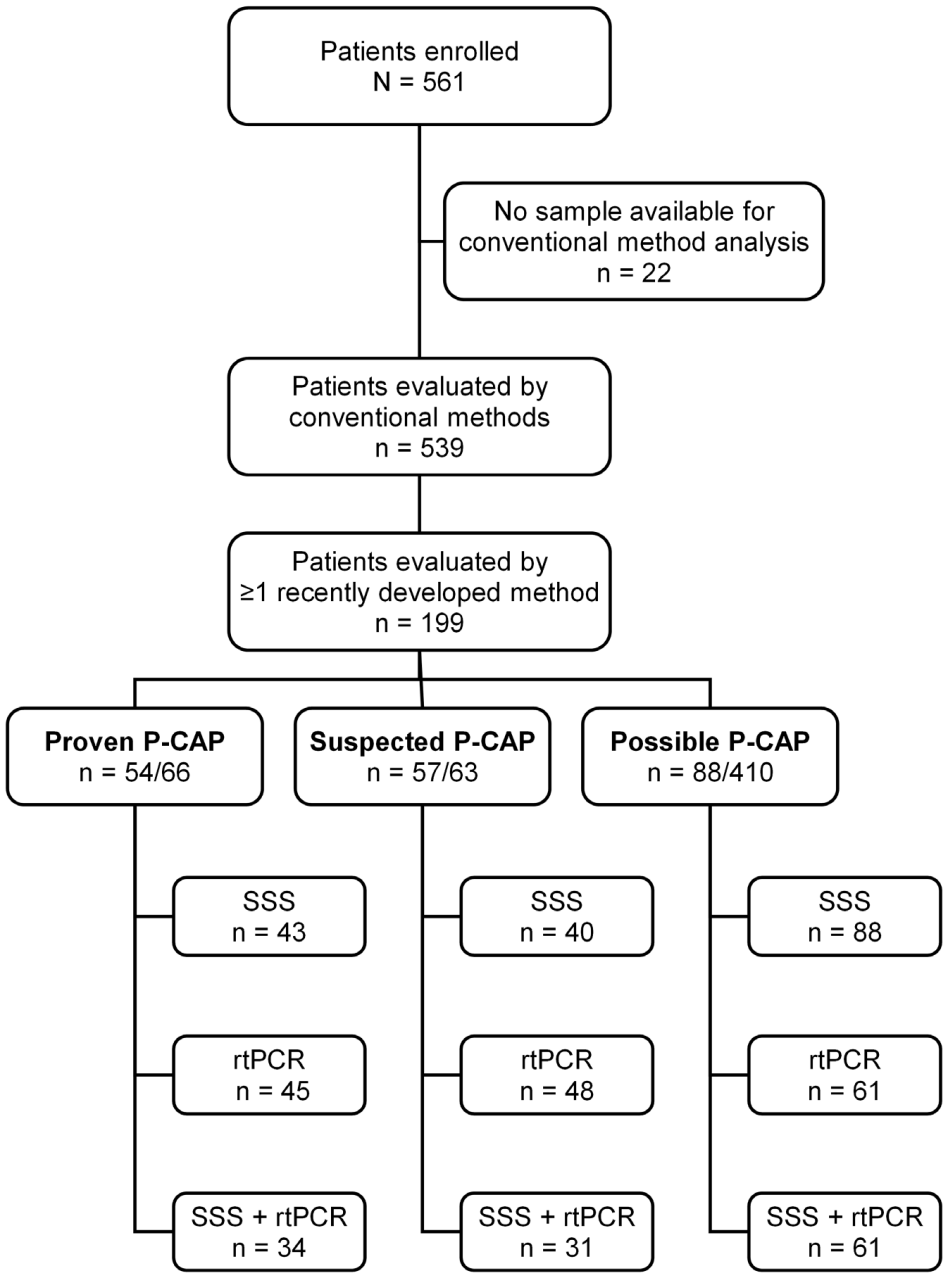
Flowchart of patients evaluated by the different detection methods. Conventional methods: blood culture, culture of pleural fluid, or polymerase chain reaction in pleural fluid. Recently developed methods: serotype-specific serology (SSS) or real-time polymerase chain reaction in blood (rtPCR). SSS included serotypes 1, 5, 6B(6D), 7F(7A), 9V(9N), 14, 19A, 19F, and 23F**.** rtPCR included serotypes 1, 3, 4, 5, 6A(6C), 6B(6D), 7F(7A), 9V(9N), 14, 18C(18B), 19A, 19F, and 23F.

The study included 561 children hospitalised in Belgium with radiologically confirmed CAP (Figure 1). The majority were boys (57.8%; p = 0.0002) and the median age was 3.6 years (IQR, 2.3–5.4 years; range, 3 months – 14 years 10 months). Forty-seven (8.4%) patients had at least one comorbidity, most of which were recurrent lower respiratory tract infections (3.7%) and asthma (1.6%). The majority (57.9%) of patients had received pneumococcal vaccination. Of these, 88% were fully vaccinated according to their age. All but five patients had received PCV7. The latter had received the 23-valent polysaccharide vaccine (Pneumo23, Sanofi Pasteur MSD).

The majority (61.1%) of patients had lobar pneumonia, followed by pleural effusion (20.0%), bronchopneumonia (17.3%), and interstitial CAP (1.6%). The median duration of symptoms before inclusion was 3 days, and 20.1% of the patients had received antibiotic treatment before inclusion.

### Aetiological diagnosis using conventional methods

A sample for blood culture, pleural fluid culture, or PCR in pleural fluid was available for 539/561 (96.1%) patients. One patient had an available pleural fluid sample but had no sample for blood culture (Table 2). P-CAP was proven in 66/539 (12.2%) patients; by positive blood culture in 50, positive pleural fluid culture in 8, and positive PCR in pleural fluid in 15 patients. Bacteria other than *S. pneumoniae* were detected in blood in 4/539 (0.7%), respiratory samples were positive for viruses in 61/193 (31.6%), and *Mycoplasma pneumoniae* was detected in 4/160 (2.5%) patients (Table 3).

**Table 2:** Number of samples available and analysed with each detection method.

	Pneumococcal conventional methods			Pneumococcal additional detection methods		Detection of other pathogens	
	Blood culture	Pleural fluid culture	Pleural fluid PCR	Blood SSS	Blood rtPCR	NPA viral culture	NPA viral and atypical PCR
Available samples, n (%)	538 (95.9)	53 (47.3) a	53 (47.3) a	383 (71.0)	519 (96.3)	193 (35.3)	193 (35.3)
Samples analysed, n (%)	538 (100.0)	49 (92.5)	22 (41.5)	171 (45.2)	154 (29.7)	163 b (82.3)	160 b (80.8)

NPA, nasopharyngeal aspirate or swab; PCR, polymerase chain reaction; rtPCR, real-time polymerase chain reaction; SSS, serotype-specific serology on paired serum samples.

a proportion of patients with pleural effusion (N = 112).

b number of samples that were analysed by each method;130/193 samples were analysed by both methods.

**Table 3:** Detected aetiologies for the study population according to age.

	Total	Age <2 yrs	Age ≥2 yrs	p-value	Age <5 yrs	Age ≥5 yrs	p-value
**Aerobic bacteria**	159/539 (29.5)	19/110 (17.3)	140/429 (32.6)	0.002	100/382 (26.2)	59/157 (37.6)	0.008
*Streptococcus pneumoniae*	332/539 (61.7) a						
Conventional methods alone	66/539 (12.2)	10 (9.1)	56 (13.1)	NS	45 (11.8)	21 (13.4)	NS
All methods combined	156/211 (73.9) b	17/28 (60.7)	139/183 (76.0)	NS	98/143 (68.5)	58/68 (85.3)	0.0095
*Streptococcus pyogenes*	3/539 (0.6)	2/110 (1.8)	1/429 (0.2)	NS	2/382 (0.5)	1/157 (0.6)	NS
Non-typeable *Haemophilus influenzae*	1/539 (0.2) c	0	1/429 (0.2)	NS	1/382 (0.3)	0	NS
*Pseudomonas aeruginosae*	1/539 (0.2) d	1/110 (0.9)	0	NS	1/382 (0.3)	0	NS
**Viruses, n tested**	193	59	134		151	42	
Virus detected	61 (31.6)	23 (39.0)	38(28.4)	NS	50 (33.1)	11 (26.2)	NS
Respiratory syncytial virus	12 (6.2)	3 (5.1)	9 (6.7)	NS	10 (6.6)	2 (4.8)	NS
Influenza A	24 (12.4)	8 (13.6)	16 (11.9)	NS	18 (11.9)	6 (14.3)	NS
Influenza B	27 (14.0)	15 (25.4)	12 (8.9)	0.01	25 (16.6)	2 (4.8)	NS
Other	2 (1.0)	1 (1.7)	1 (0.7)	-	1 (0.7)	1 (2.4)	-
***Mycoplasma pneumoniae,*** ** n tested**	160	52	108		127	33	
Yes	4 (2.5)	0	4 (3.7)	NS	0	4 (12.1)	0.002

Values are numbers (%) of subjects. NS, not significant.

a All detection methods combined and adjusted for the whole study population.

b Ratio of all patients with pneumococcal pneumonia based on any of the applied detection methods/ all patients with proven P-CAP (N = 66), and those with suspected P-CAP (N = 57) and possible P-CAP (N = 88) that were analysed by at least one new detection method.

c Found in combination with *S. pneumoniae.*

d Found in combination with *S. pyogenes.*

### Detection of pneumococcal aetiology using SSS or rtPCR in blood in patients with proven, suspected, or possible P-CAP

Of the 539 patients included in the conventional analysis, 66 had proven P-CAP and 63 had suspected P-CAP. Disease characteristics were similar in the three groups except for CAP type, CRP concentration, and antibiotic use before admission (Table 4). At least one additional detection method was performed in 199 patients: SSS in 171 patients, rtPCR in blood in 154 patients, and both SSS and rtPCR in blood in 126 patients (Figure 1, Table 2).

**Table 4:** Patient and disease characteristics for all patients with proven and suspected P-CAP and the subgroup of patients with possible P-CAP analysed by at least one new detection method (N = 217).

	Proven P-CAP (N = 66)	Suspected P-CAP (N = 63)	Possible P-CAP (N = 88)	p-value
Age (yrs), median (IQR)	4.0 (2.7–5.3)	4.0 (2.7–7.1)	3.9 (2.7–5.9)	NS
Sex (F/M)	27/39	29/34	41/47	NS
Inflammatory parameters, median (IQR)				
WBC count (×10^9^/L)	18.6 (14.2–27.6)	15.9 (12.1–22.5)	19.0 (14.5–25.1)	NS
Neutrophils (%)	80.0 (72.9–86.3)	78.5 (64.8–84.9)	80.0 (69.7–84.7)	NS
CRP (mg/L)	244.5 (175.0–374.0)	279.0 (195.0–352.0)	170.0 (74.5–258.0)	<0.0001
CAP type, n (%)				
Lobar CAP	26 (39.4)	0	69 (78.4)	<0.0001
Bronchopneumonia	3 (4.5)	0	16 (18.2)	0.0002
Pleural effusion	36 (54.5)	63 (100)	1 (1.1)	<0.0001
Interstitial CAP	1 (1.5)	0	2 (2.3)	NS
Antibiotic treatment before admission, n (%)	7 (10.6)	20 (31.7)	8 (9.1)	0.0003
Vaccination with PCV7, n (%)	36 (54.5)	35 (55.5)	53 (60.2)	NS
Comorbidity, n (%)	6 (9.1)	5 (7.9)	9 (10.2)	NS

CAP, community-acquired pneumonia; CRP, C-reactive protein; IQR, interquartile range; P-CAP, pneumococcal community-acquired pneumonia; PCV7, 7-valent pneumococcal conjugate vaccine; WBC, white blood cells.

Of the 126 patients that had both SSS and rtPCR in blood analysis, SSS was positive in 75 (59.5%) and rtPCR in blood was positive in 37 (29.4%), whereas the conventional methods were positive in 34 (27.0%) (Table 5). Results of SSS and rtPCR in blood agreed in 66 (52.4%) patients. SSS was positive for 49 patients that were rtPCR in blood-negative.

**Table 5:** Comparison of SSS and rtPCR in blood results for the 126 patients evaluated by both additional methods.

Detection of *S. pneumoniae*	Proven P-CAP	Suspected P-CAP	Possible P-CAP	Total	Total adjusted for whole study population (%)
Blood culture, pleural fluid culture, or pleural fluid PCR	34	-	-	34/126 (27.0)	12.2
SSS-IgG or SSS-IgA a	29/34 (85.3)	16/31 (51.6)	30/61 (49.2)	75/126 (59.5)	53.9
rtPCR in blood b	18/34 (52.9)	16/31 (51.6)	3/61 (4.9)	37/126 (29.4)	16.3

Values are numbers (%) of subjects. P-CAP, pneumococcal community-acquired pneumonia; PCR, polymerase chain reaction; rtPCR, real-time polymerase chain reaction; SSS, serotype-specific serology.

a SSS included serotypes 1, 5, 6B(6D), 7F(7A), 9V(9N), 14, 19A, 19F, and 23F.

b rtPCR in blood included serotypes 1, 3, 4, 5, 6A(6C), 6B(6D), 7F(7A), 9V(9N), 14, 18C(18B), 19A, 19F, and 23F.

Compared to conventional methods, the additional diagnostic value was 36.5% for SSS and 15.1% for rtPCR in blood.

Previous antibiotic treatment did not change positivity rates of SSS and rtPCR in blood but it reduced that of blood culture (47/435 [10.8%] in the untreated group vs. 3/103 [2.9%] in the treated group, p = 0.013). In addition, rtPCR in blood was more often positive in patients with pleural effusion than in those with isolated lobar CAP (p<0.0001), and in patients with suspected or proven P-CAP than in patients with possible P-CAP (p<0.0001).

Combining the results of all 66 proven P-CAP patients and the 145 non-proven P-CAP patients (including 43/57 [75.4%] with suspected P-CAP, and 47/88 [53.4%] with possible P-CAP) that were evaluated by conventional methods and at least one additional detection method indicated pneumococcal aetiology in 156/211 (73.9%) patients. This corresponded to 61.7% of CAP cases when adjusted to the entire study population.

Diagnosis of pneumococcal aetiology was associated with age ≥5 years (85.3% vs. 68.5%; p = 0.0095; Table 3), pleural effusion, a higher CRP concentration, a higher percentage of neutrophils in the peripheral blood, admission to an intensive care unit, chest tube drainage, and mechanical ventilation (data not shown).

### Pneumococcal serotype distribution

**Figure 2:**
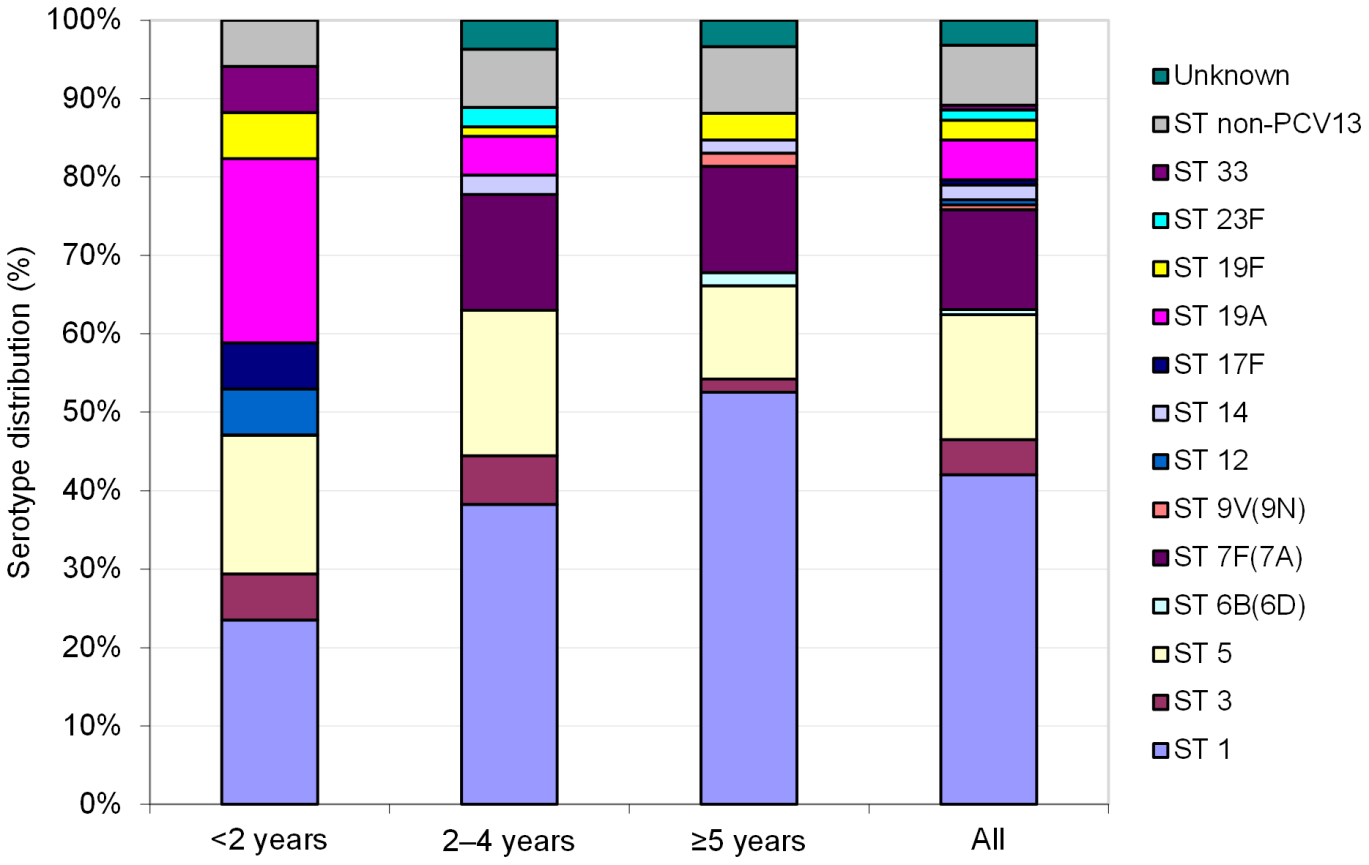
Serotype distribution in all patients with pneumococcal infection. Diagnosis was based on any of the assays (N = 156). The causative serotype for a patient was determined according to the following hierarchy of assays: Quellung reaction on pneumococcal isolates > rtPCR in blood > SSS-IgG > SSS-IgA. rtPCR, real-time polymerase chain reaction in blood; SSS, serotype-specific serology; ST, serotype. Non-PCV13, serotypes not included in 13-valent pneumococcal conjugate vaccine as determined by rtPCR in blood. SSS included serotypes 1, 5, 6B(6D), 7F(7A), 9V(9N), 14, 19A, 19F, and 23F**.** rtPCR in blood included serotypes 1, 3, 4, 5, 6A(6C), 6B(6D), 7F(7A), 9V(9N), 14, 18C(18B), 19A, 19F, and 23F. One patient had both serotypes 1 and 5 detected by rtPCR in blood.

The causative serotype was determined by the Quellung reaction for 44/156 (28.2%), by rtPCR in blood for 42 (26.9%), by SSS-IgG for 58 (37.2%), and by SSS-IgA for 7 (4.5%) patients. The causative serotype was unknown for 5/156 (3.2%) patients. According to the Quellung reaction alone, serotype 1 was the most frequent, followed by serotypes 5, 19A, 3, 7F(7A), 12, 17F, and 33. Among the serotypes tested using any method, serotype 1 was also the most frequent (42.3%), followed by serotypes 5, 7F(7A), 19A, and 3 (Figure 2). In one patient, serotypes 1 and 5 were detected simultaneously by rtPCR in blood. Serotype 1 infection was associated with an age ≥5 years (31/58 [53.4%] in ≥5 year-olds versus 33/98 [33.7%] in <5 year-olds; p = 0.03). In contrast, serotype 19A was never detected in children ≥5 years-old and was associated with an age <2 years (4/17 [23.5%] in <2 year-olds versus 4/139 [2.9%] in ≥2 year-olds; p = 0.0053).

Serotype 1 was found more frequently in patients with lobar CAP (33/65 [50.8%]) than in patients with bronchopneumonia or pleural effusion (31/91 [34.1%], p = 0.026). Infection with serotypes 1, 3, 5, or 19A was not associated with either complicated P-CAP (presence of pleural effusion) or invasive P-CAP (complicated or proven P-CAP). In contrast, serotype 7F(7A) was found more in patients with non-proven P-CAP (18/90 [20%]) compared to patients with proven P-CAP (2/66 [3.0%], p = 0.0017) and compared to patients with non-invasive P-CAP (p = 0.03).

PCV7-serotypes were detected in 11 patients of whom three were completely vaccinated with PCV7; one 4-year-old patient with proven P-CAP (PCR in pleural fluid positive and serotype 14 detected by SSS) and two patients (8 years and 4 years of age) with possible P-CAP (serotypes 19F and 23F detected by SSS). Of the remaining eight patients, only one received a single dose of PCV7, the others were not vaccinated.

## Discussion

This is the first study that evaluated pneumococcal aetiology and serotype distribution in children hospitalised because of CAP after the implementation of PCV7 vaccination for infants and children ≤2 years of age in Belgium. To maximise the yield of pneumococcal aetiological diagnosis, a combination of conventional methods (blood culture, pleural fluid culture, and PCR in pleural fluid) and additional detection methods (rtPCR in blood, SSS-IgG, and SSS-IgA) were used. Conventional methods established pneumococcal aetiology in 12.2% of the patients but combination of all detection methods indicated pneumococcal aetiology in 61.7% of the whole study population. These rates are higher than previously reported (14.3%–46%) [3]–[5], [14]–[18]. These results underline the importance of routine use of conventional methods and confirm previous findings that rtPCR in blood and SSS are more sensitive than blood culture for identifying pneumococcal aetiology in childhood CAP. Previous antibiotic treatment may contribute to this difference because it reduced the sensitivity of blood culture but not that of rtPCR in blood or SSS.

In our study, SSS had a high positivity rate in non-invasive CAP, whereas both rtPCR in blood and SSS had high positivity rates in complicated non-proven P-CAP. The positivity rate of SSS in 52.3% of non-invasive CAP cases supports the finding of others that serology may detect less severe cases than blood culture [4], [16]. The accuracy of this finding is also supported by the demonstrated effect of PCV7 and other PCVs on reduction of overall CAP, which also suggests that a substantial number of non-invasive CAP cases are related to pneumococcal infection [6], [7].

Before PCV7 introduction in Belgium, about 60% of proven pneumococcal CAP cases in children <5 years-old (65% in <2 years-olds and 57% in 2–4 years-olds) were due to PCV7 serotypes [8]. At that time, serotypes 14, 1, 6B and 23F were most frequent; serotype 14 predominated in children <2 years old whereas serotype 1 predominated in children 2-<5 years old [11]. As previously reported for IPD and parapneumonic effusion [18]–[21], we found that, in a well-vaccinated cohort of children, non-PCV7 serotypes accounted for the majority (92.2%) of P-CAP cases, of which 73.1% are theoretically covered by the licensed 10-valent PCV (Synflorix, GlaxoSmithKline Vaccines) and 83.4% by the 13-valent PCV (Prevenar 13, Pfizer, Inc.). Our results thus indicate that increased serotype coverage is needed to further address the burden of childhood CAP in Belgium. Since September 2011, PCV7 has been replaced by PCV13 in the Belgian childhood vaccination schedule. The impact of PCV13 on childhood CAP and in particular on invasive or complicated childhood CAP is currently unknown.

Using combined serotype detection methods, we found that serotypes 1, 5, 7F(7A), 19A, and 3 were the most common in childhood CAP in a population that was well vaccinated with PCV7. Few authors have reported serotype distribution in CAP, and most of them reported only complicated parapneumonic effusion [5], [15], [18], [19], [22]. We found the same five predominant serotypes as in most studies, although the relative proportions differed. The finding that serotype 1 was the most common agrees with previous reports [5], [8], [11], [18], [23]. For the first time, serotype 5 was the second-most common in CAP, although it has been consistently found in invasive pneumococcal disease (IPD) and CAP [5], [8], [11], [23]. Serotype 7F(7A) is also frequently found in IPD and parapneumonic effusion [19]. Serotypes 19A and 3 are increasingly found in PCV7-vaccinated populations and are associated with complicated CAP [5], [18], [22], [24]. We found these two serotypes in approximately 5% of patients, less than usually reported for parapneumonic effusion, CAP, or IPD (2%–27%) [5], [8], [15], [18], [19], [24], [25]. Because serotype 3 was not tested with SSS, some patients whose blood was not tested with rtPCR might have been missed. The known association of serotype 19A with an age <2 years [8], [16], also found in our study, may have contributed to its rather low detection rate, as only 20.7% of children in our study were <2 years-old. Furthermore, other factors such as antibiotic consumption and secular trends may have influenced the prevalence of serotype 19A [8], [26].

Pneumococcal aetiology was associated with complicated CAP, although in contrast to previous findings [5], [23], complicated and invasive CAP were not associated with any specific serotype. This may have been due to the higher sensitivity of SSS in non-invasive P-CAP cases compared to conventional methods and rtPCR in blood. SSS was positive for serotypes 1 or 5 in 51% of children with non-invasive P-CAP.

PCV7-serotypes were detected in three completely vaccinated and otherwise healthy children. IPD in vaccinated children is rare but has been previously reported [27], [28]. In our study, only one patient had invasive disease (pneumonia with pleural effusion) and two had non-invasive CAP. In all cases, the episode occurred more than 2 years after the last vaccine dose. Assuming that SSS reflects true infection and given the time that elapsed since the last vaccine dose, these three episodes are probably attributable to a natural decline in specific antibody concentration beneath the protective antibody threshold for CAP, which is probably higher than for IPD [12].

In contrast to other reports showing similar pneumococcal infection rates in different age groups [5], [16], [17], we found that pneumococcal aetiology was associated with an age ≥5 years. The predominance of serotype 1 may have contributed to this finding because it has been associated with an age ≥5 years [5], [11], [23]. The use of SSS-IgG may also have contributed to this difference, as we previously showed that SSS-IgG positivity rate was higher in children aged ≥2 years [12]. Finally, vaccination with PCV7 may also have contributed to this age distribution because PCV7 coverage for P-CAP in children aged <2 years was previously demonstrated to be substantially higher than in older children [23]. PCV7 vaccination may therefore have prevented relatively more cases in children aged < 2 years.

In contrast to previous studies [4], [5], our study evaluated SSS and rtPCR in blood in a large group of patients with proven pneumococcal infection. However, the interpretation of the results is limited because we selected the patients that had been evaluated with additional detection methods, which might be the most severe cases. Indeed, 59.4% of the patients evaluated by SSS or rtPCR in blood had bacteraemic or complicated CAP, with a pneumococcal detection rate of 30.4% by conventional methods compared to 12.2% in the whole study population. However, our adjustment of results to the whole study population minimises this bias.

With SSS, in contrast to culture or rtPCR, bacteria are not directly but indirectly detected through a specific immune response. Thus, the SSS high positivity rate might be due to nasopharyngeal carriage. However, strong immune responses were found in two sera taken 3–4 weeks apart and the predominant serotypes 1 and 5 are rarely found in carriage but are highly invasive [29]. Therefore, our results probably reflect true infections.

In conclusion, our study confirmed that *S. pneumoniae* remains the leading bacterial pathogen in children hospitalised with CAP since the introduction of PCV7 and *Haemophilus influenzae* type b universal infant vaccinations. Adding SSS and rtPCR in blood to conventional methods increased the detection of pneumococcal aetiology, especially for non-invasive CAP. rtPCR in blood is a practical method for improving pneumococcal detection, especially in children previously treated with antibiotics, whereas SSS is too complex for routine use but is a promising tool for epidemiological studies. Furthermore, our study indicated that PCV7-serotypes are uncommon in CAP in well-immunised children. The current serotype distribution, with predominance of serotypes 1, 5, and 7F(7A), supports the use of higher-valent pneumococcal vaccines to reduce the remaining burden of childhood CAP and address the increasing incidence of complicated CAP.

### Scientific presentations

This study was presented in part at the 28^th^ and 29^th^ annual meetings of the European Society for Pediatric Infectious Diseases in Nice, France in May 2010 and in the Hague, The Netherlands in June 2011, and the 8^th^ International Symposium on Pneumococci and Pneumococcal Diseases, Iguaçu Falls, Brazil in March 2012.

## Supporting Information

Text S1 **Ethics committees involved.**
(docx)

## References

[1] McIntosh K (2002) Community-acquired pneumonia in children. N Engl J Med 346: 429–437. 11832532 10.1056/NEJMra011994

[2] Harris M, Clark J, Coote N, Fletcher P, Harnden A, et al (2011) British Thoracic Society guidelines for the management of community acquired pneumonia in children: update 2011. Thorax 66 Suppl 2ii1–23. 21903691 10.1136/thoraxjnl-2011-200598

[3] Cevey-Macherel M, Galetto-Lacour A, Gervaix A, Siegrist CA, Bille J, et al (2009) Etiology of community-acquired pneumonia in hospitalized children based on WHO clinical guidelines. Eur J Pediatr 168: 1429–1436. 19238436 10.1007/s00431-009-0943-y PMC7087130

[4] Esposito S, Bosis S, Cavagna R, Faelli N, Begliatti E, et al (2002) Characteristics of Streptococcus pneumoniae and atypical bacterial infections in children 2-5 years of age with community-acquired pneumonia. Clin Infect Dis 35: 1345–1352. 12439797 10.1086/344191

[5] Resti M, Moriondo M, Cortimiglia M, Indolfi G, Canessa C, et al (2010) Community-acquired bacteremic pneumococcal pneumonia in children: diagnosis and serotyping by real-time polymerase chain reaction using blood samples. Clin Infect Dis 51: 1042–1049. 20883110 10.1086/656579

[6] Elemraid MA, Rushton SP, Shirley MD, Thomas MF, Spencer DA, et al. (2012) Impact of the 7-valent pneumococcal conjugate vaccine on the incidence of childhood pneumonia. Epidemiol Infect: 1–8. 10.1017/S0950268812002257 PMC3733065 23084696

[7] Grijalva CG, Nuorti JP, Arbogast PG, Martin SW, Edwards KM, et al (2007) Decline in pneumonia admissions after routine childhood immunisation with pneumococcal conjugate vaccine in the USA: a time-series analysis. Lancet 369: 1179–1186. 17416262 10.1016/S0140-6736(07)60564-9

[8] Hanquet G, Lernout T, Vergison A, Verhaegen J, Kissling E, et al (2011) Impact of conjugate 7-valent vaccination in Belgium: addressing methodological challenges. Vaccine 29: 2856–2864. 21342667 10.1016/j.vaccine.2011.02.016

[9] Cherian T, Mulholland EK, Carlin JB, Ostensen H, Amin R, et al (2005) Standardized interpretation of paediatric chest radiographs for the diagnosis of pneumonia in epidemiological studies. Bull World Health Organ 83: 353–359. 15976876 PMC2626240

[10] Corless CE, Guiver M, Borrow R, Edwards-Jones V, Fox AJ, et al (2001) Simultaneous detection of *Neisseria meningitidis*, *Haemophilus influenzae*, and *Streptococcus pneumoniae* in suspected cases of menigitis and septicemia using real-time PCR. J. Clin. Microbiol. 39(4): 1553–1558. 10.1128/JCM.39.4.1553-1558.2001 PMC87969 11283086

[11] Vergison A, Tuerlinckx D, Verhaegen J, Malfroot A (2006) Belgian Invasive Pneumococcal Disease Study Group (2006) Epidemiologic features of invasive pneumococcal disease in Belgian children: passive surveillance is not enough. Pediatrics 118: e801–809. 16894008 10.1542/peds.2005-3195

[12] Tuerlinckx D, Smet J, De Schutter I, Jamart J, Vergison A, et al (2013) Evaluation of a WHO-Validated Serotype-Specific Serological Assay for the Diagnosis of Pneumococcal Etiology in Children with Community-Acquired Pneumonia. Pediatr Infect Dis J 32: e277–284. 23407099 10.1097/INF.0b013e31828c363f

[13] Albrich WC, Madhi SA, Adrian PV, van Niekerk N, Mareletsi T, et al (2012) Use of a rapid test of pneumococcal colonization density to diagnose pneumococcal pneumonia. Clin Infect Dis 54: 601–609. 22156852 10.1093/cid/cir859 PMC3275757

[14] Don M, Fasoli L, Paldanius M, Vainionpaa R, Kleemola M, et al (2005) Aetiology of community-acquired pneumonia: serological results of a paediatric survey. Scand J Infect Dis 37: 806–812. 16308213 10.1080/00365540500262435

[15] Esposito S, Marchese A, Tozzi AE, Rossi GA, Dalt LD, et al (2012) Bacteremic Pneumococcal Community-acquired Pneumonia in Children Less Than 5 Years of Age in Italy. Pediatr Infect Dis J 31: 705–710. 22426300 10.1097/INF.0b013e31825384ae

[16] Juven T, Mertsola J, Waris M, Leinonen M, Meurman O, et al (2000) Etiology of community-acquired pneumonia in 254 hospitalized children. Pediatr Infect Dis J 19: 293–298. 10783017 10.1097/00006454-200004000-00006

[17] Michelow IC, Olsen K, Lozano J, Rollins NK, Duffy LB, et al (2004) Epidemiology and clinical characteristics of community-acquired pneumonia in hospitalized children. Pediatrics 113: 701–707. 15060215 10.1542/peds.113.4.701

[18] Elemraid MA, Sails AD, Eltringham GJ, Perry JD, Rushton SP, et al. (2013) Aetiology of paediatric pneumonia after the introduction of pneumococcal conjugate vaccine. Eur Respir J. 10.1183/09031936.00199112 PMC3844138 23598951

[19] Obando I, Munoz-Almagro C, Arroyo LA, Tarrago D, Sanchez-Tatay D, et al (2008) Pediatric parapneumonic empyema, Spain. Emerg Infect Dis 14: 1390–1397. 18760005 10.3201/eid1409.071094 PMC2603109

[20] Hsu KK, Shea KM, Stevenson AE, Pelton SI (2010) Changing serotypes causing childhood invasive pneumococcal disease: Massachusetts, 2001-2007. Pediatr Infect Dis J 29: 289–293. 19935447 10.1097/INF.0b013e3181c15471

[21] Whitney CG, Farley MM, Hadler J, Harrison LH, Bennett NM, et al (2003) Decline in invasive pneumococcal disease after the introduction of protein-polysaccharide conjugate vaccine. N Engl J Med 348: 1737–1746. 12724479 10.1056/NEJMoa022823

[22] Byington CL, Korgenski K, Daly J, Ampofo K, Pavia A, et al (2006) Impact of the pneumococcal conjugate vaccine on pneumococcal parapneumonic empyema. Pediatr Infect Dis J 25: 250–254. 16511389 10.1097/01.inf.0000202137.37642.ab

[23] De Schutter I, Malfroot A, Pierard D, Lauwers S (2006) Pneumococcal serogroups and serotypes in severe pneumococcal pneumonia in Belgian children: theoretical coverage of the 7-valent and 9-valent pneumococcal conjugate vaccines. Pediatr Pulmonol 41: 765–770. 16779850 10.1002/ppul.20437

[24] Byington CL, Hulten KG, Ampofo K, Sheng X, Pavia AT, et al (2010) Molecular epidemiology of pediatric pneumococcal empyema from 2001 to 2007 in Utah. J Clin Microbiol 48: 520–525. 20018815 10.1128/JCM.01200-09 PMC2815589

[25] Kaplan SL, Barson WJ, Lin PL, Stovall SH, Bradley JS, et al (2010) Serotype 19A Is the most common serotype causing invasive pneumococcal infections in children. Pediatrics 125: 429–436. 20176669 10.1542/peds.2008-1702

[26] Hausdorff WP, Hoet B, Schuerman L (2010) Do pneumococcal conjugate vaccines provide any cross-protection against serotype 19A? BMC Pediatr 10: 4. 20122261 10.1186/1471-2431-10-4 PMC2829470

[27] Harboe ZB, Valentiner-Branth P, Ingels H, Rasmussen JN, Andersen PH, et al (2013) Pediatric invasive pneumococcal disease caused by vaccine serotypes following the introduction of conjugate vaccination in Denmark. PLoS One 8: e51460. 23365635 10.1371/journal.pone.0051460 PMC3554759

[28] O'Brien KL, Moisi J, Romero-Steiner S, Holder P, Carlone GM, et al (2009) Pneumococcal antibodies in a child with type 14 pneumococcal conjugate vaccine failure. Vaccine 27: 1863–1868. 19171175 10.1016/j.vaccine.2008.12.060

[29] Weinberger DM, Harboe ZB, Sanders EA, Ndiritu M, Klugman KP, et al (2010) Association of serotype with risk of death due to pneumococcal pneumonia: a meta-analysis. Clin Infect Dis 51: 692–699. 20715907 10.1086/655828 PMC2927802

