# Supplementary material for: Pneumococcal Aetiology and Serotype Distribution in Paediatric Community-Acquired Pneumonia
Source: PLoS One. 2014 Feb 18;9(2):e89013. doi: 10.1371/journal.pone.0089013 (PMC3928328; doi:10.1371/journal.pone.0089013)
Supplement: Text S1 — Ethics committees involved. (docx) [file pone.0089013.s001.docx]

**Text S1. Ethics committees involved**

**List of ethics committees that approved the study protocol**

- Leading ethics committee:
- Universitair Ziekenhuis Brussel (UZ Brussel), Laarbeeklaan 101, 1090 Brussel
- Local ethics committees:
- ASZ Aalst, Merestraat 80, 9300 Aalst
- AZ St. Jan A.V., Ruddershove 10, 8000 Brugge
- CH Jolimont-Lobbes, Rue Ferrer 159, 7100 Haine St. Paul
- CHR de la Citadelle, Bld. du 12ieme de Ligne 1, 4000 Liège
- CHU de Charleroi, Rue de Gozée 706, 6110 Montigny-Le-Tilleul
- CHU Tivoli, Av. Max Buset 34, 7100 La Louvière
- Cliniques du Sud-Luxembourg, Rue des Déportées 137, 6700 Arlon
- G.V.A-St. Vincentiusziekenhuis, St. Vincentiusstraat 20, 2018 Antwerpen
- G.V.A- AZ St. Augustinus, Oosterveldlaan 24, 2610 Wilrijk
- Hôpitaux RIS Sud Etterbeek, Rue Marconi 142, 1190 Bruxelles
- HUDERF, Av. G. G. Croq 15, 1020 Bruxelles
- Serruys Ziekenhuis A.V., Kaïrostraat 84, 8400 Oostende
- UCL Mont Godinne, Av. de Dr. Therasse 1, 5530 Yvoir
- UCL St. Luc, Av. Hippocrate 55-14, 1200 Bruxelles
- UZ Antwerpen, Wilrijkstraat 10, 2650 Edegem
- UZ Gent, De Pintelaan 185.2P4, 9000 Gent
- Virga Jesse Ziekenhuis, Stadsomvaart 11, 3500 Hasselt
- ZNA Middelheim, Lindendreef 1, 2020 Antwerpen
- AZ Klina, Augustijnlei 100, 2930 Brasschaat
- CHR Namur, Av. Albert Ier 185, 5000 Namur
- CHC Liège, Rue de Hesbaye 75, 4000 Liège
